# P-1636. Comparative Effectiveness of Combination Therapy with Nirmatrelvir-Ritonavir and Molnupiravir versus Monotherapy with Molnupiravir or Nirmatrelvir-Ritonavir in Hospitalised COVID-19 Patients: A Target Trial Emulation Study

**DOI:** 10.1093/ofid/ofaf695.1812

**Published:** 2026-01-11

**Authors:** Ming Hong Choi, Eric Yuk Fai Wan, Fan Ngai Ivan Hung

**Affiliations:** Queen Mary Hospital, Hong Kong, Hong Kong; The University of Hong Kong, Hong Kong, Not Applicable, Hong Kong; The University of Hong Kong, Hong Kong, Not Applicable, Hong Kong

## Abstract

**Background:**

While molnupiravir and nirmatrelvir-ritonavir have demonstrated efficacy in reducing hospitalisation and mortality among unvaccinated, high-risk COVID-19 patients in outpatient settings, their impact on hospitalised adults remains unclear. Preclinical studies and case reports suggest combining these antivirals may reduce viral shedding and enhance survival.

**Figure:**
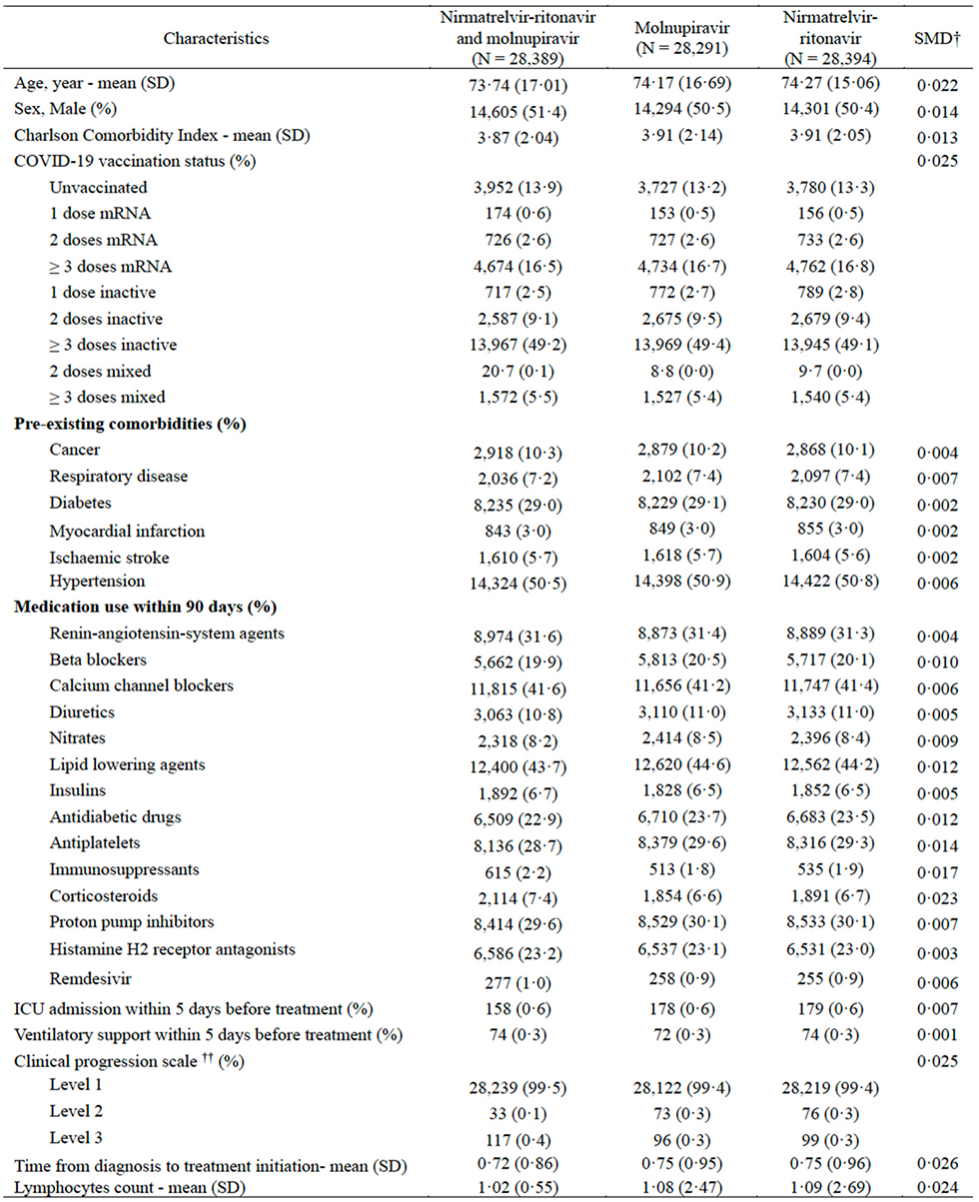
Baseline characteristics of eligible COVID-19 patients after the inverse probability of treatment weighting (IPTW) SMD=Standardised mean difference; SD=Standard deviation; IQR = interquartile range; CCI=Charlson Comorbidity Index; ICU=Intensive care units; †SMD<0.1 indicates balance between groups· †† Level 1: Hospitalised patients with no oxygen therapy; Level 2: Hospitalised patients with oxygen by mask, nasal prongs, non-invasive ventilation or high flow; Level 3: Hospitalised patients with intubation and mechanical ventilation, vasopressors, dialysis, or extracorporeal membrane oxygenation

**Figure:**
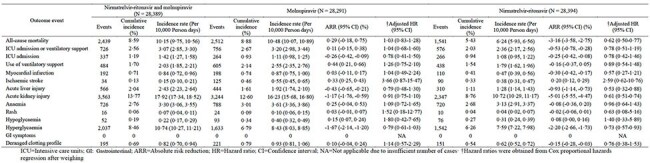
Risk of outcomes for COVID-19 patients receiving combined use of molnupiravir and nirmatrelvir ritonavir compared with patients receiving molnupiravir alone and patients receiving nirmatrelvir-ritonavir alone after weighting

**Methods:**

This target trial emulation study evaluated the safety and efficacy of combined molnupiravir and nirmatrelvir-ritonavir versus monotherapy in hospitalised COVID-19 adults in Hong Kong. Data were extracted from electronic health records of patients aged 18 and older treated within five days of hospital admission between March 16, 2022, and March 31, 2024. Inverse probability of treatment weighting (IPTW) was used to balance baseline characteristics. Outcomes, including all-cause mortality, Intensive Care Unit (ICU) admission, and ventilatory support, were assessed using Cox proportional hazards models.

**Figure:**
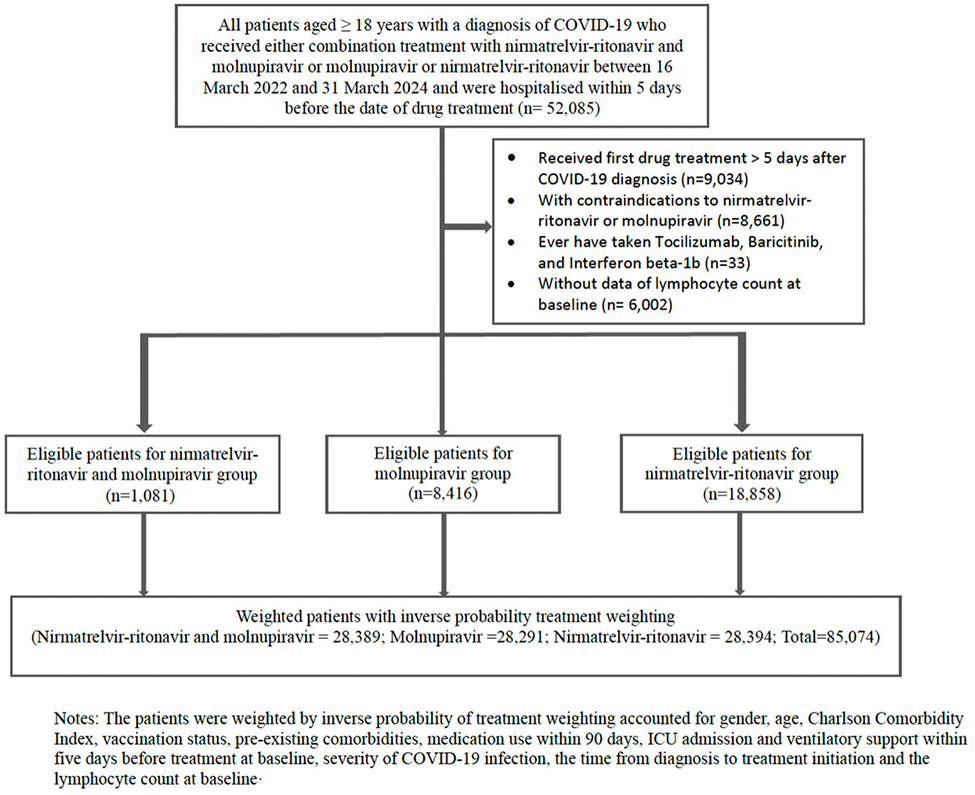
Study flow diagram 90-day cumulative incidence of outcomes in recipients of combination treatment with nirmatrelvir-ritonavir and molnupiravir compared to recipients of molnupiravir monotherapy and recipients of nirmatrelvir-ritonavir

**Figure:**
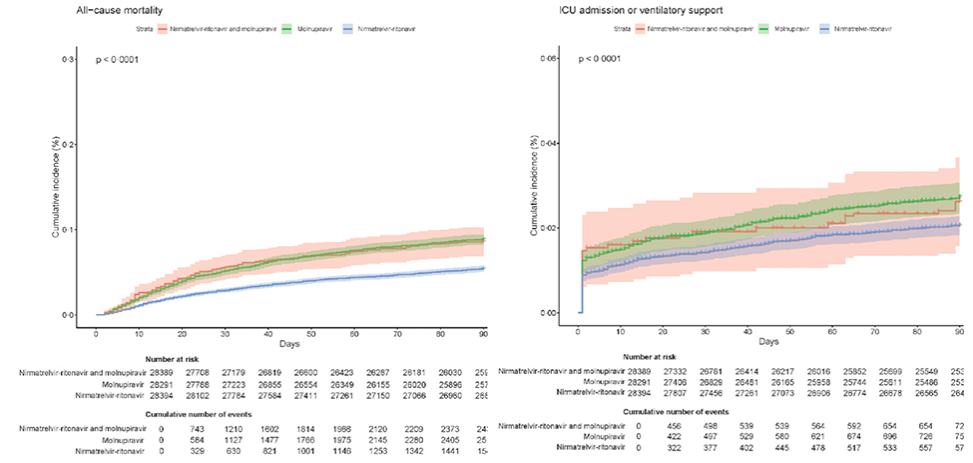
Shared area refers to the 95% confidence interval for the cumulative incidence. The P values indicate the overall P values of the Log-rank test comparing the three treatment groups for each outcome

**Results:**

Among 28,355 patients (combination: 1,081; molnupiravir: 8,416; nirmatrelvir-ritonavir: 18,858), IPTW-adjusted analyses showed that nirmatrelvir-ritonavir monotherapy was associated with a significantly lower risk of mortality (HR: 0.62; 95% CI 0.50-0.77; ARR: -3.16%) compared to combination therapy. Risks of ICU admission and ventilatory support were similar across all groups. Patients receiving nirmatrelvir-ritonavir monotherapy also showed lower risks of acute liver injury (HR: 0.53 [95% CI 0.32-0.88]), acute kidney injury (HR: 0.61 [95% CI 0.51-0.74]), and hyperglycaemia (HR 0·73 [95% CI 0.57- 0.93]).

**Conclusion:**

Combining nirmatrelvir-ritonavir and molnupiravir does not significantly reduce mortality, ICU admissions, or ventilatory support needs in hospitalised COVID-19 adults. Further randomised controlled trials are needed to confirm these findings.

**Disclosures:**

All Authors: No reported disclosures

